# Tracheostomy in high-risk patients on ECMO: A bedside hybrid dilational technique utilizing a Rummel tourniquet

**DOI:** 10.1016/j.sopen.2023.11.010

**Published:** 2023-11-20

**Authors:** Britton B. Donato, Marisa Sewell, Megan Campany, Ga-ram Han, Taylor S. Orton, Marko Laitinen, Jacob Hammond, Xindi Chen, Jasmina Ingersoll, Ayan Sen, Jonathan D'Cunha

**Affiliations:** a Mayo Clinic, Department of Surgery, Phoenix, AZ, United States of America; b Medical College of Wisconsin, Division of Cardiothoracic Surgery, Milwaukee, WI, United States of America; c Oregon Health and Science University, Department of General Surgery, Portland, OR, United States of America; d Mayo Clinic Alix School of Medicine, Scottsdale, AZ, United States of America; e Mayo Clinic, Department of Critical Care Medicine, Phoenix, AZ, United States of America; f Mayo Clinic, Department of Cardiothoracic Surgery, Phoenix, AZ, United States of America

**Keywords:** Tracheostomy, Rummel tourniquet, ECMO, Anticoagulation

## Abstract

**Objective:**

Traditionally, critically ill patients requiring prolonged mechanical ventilation benefit from a long-term airway, thus necessitating tracheostomy. The widespread application of extracorporeal membrane oxygenation (ECMO) has exponentially increased in recent years, creating a new subset of patients necessitating tracheostomy with significantly increased bleeding risk. We present a hybrid dilational tracheostomy technique utilizing a Rummel tourniquet developed at our institution to mitigate bleeding risk in patients on ECMO necessitating long-term airway.

**Methods:**

A total of 24 patients on ECMO underwent bedside hybrid dilational tracheostomy with utilization of a Rummel tourniquet from 06/2020 to 01/2022 at our institution. These patients were followed longitudinally and evaluated for postoperative bleeding. Particular attention was paid to anticoagulation regimens pre- and post-operatively.

**Results:**

The primary outcome of the study, postoperative bleeding, was observed in four of the 24 study participants (16.67 %). Each of these complications were managed with tightening of the Rummel tourniquet and application of hemostatic packing agents; no operative interventions were required. Anticoagulation was held for a mean time of 4.33 h preoperatively and 5.2 h postoperatively.

**Conclusions:**

Our data support this hybrid tracheostomy technique with the addition of a Rummel tourniquet to be a safe and effective adjunct for perioperative hemostasis in high-risk patients necessitating tracheostomy while on ECMO. While this technique was initially developed for critically ill COVID-19 patients, we believe it can be applied to all patients on ECMO to help mitigate perioperative bleeding risk.

## Highlights

• Patients on ECMO necessitating tracheostomy have an increased bleeding risk.
• A Rummel tourniquet can be utilized to minimize post-tracheostomy bleeding.
• Our data demonstrate this technique to be safe and effective.

## Graphical abstract

**Figure:**
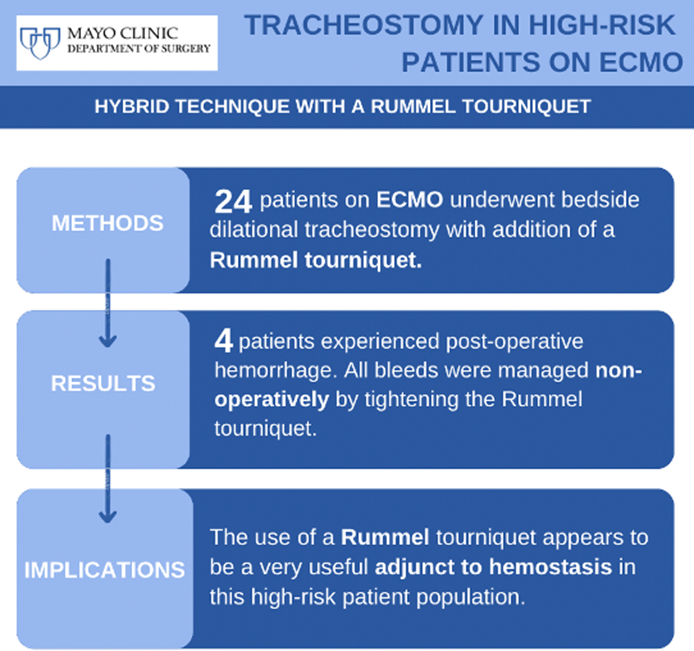

## Central picture

**Figure:**
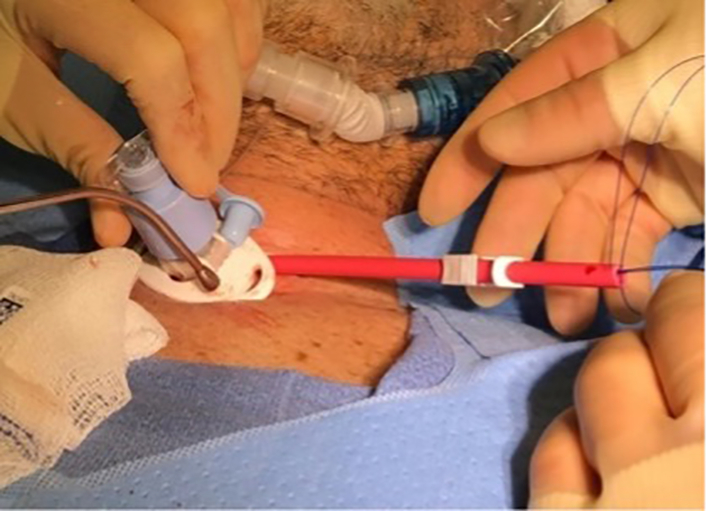

Central Picture Legend: Placement of pursestring suture on a Rummel tourniquet around tracheostomy stoma.

## Central message

Our data support a hybrid tracheostomy technique with addition of a Rummel tourniquet is a safe and effective adjunct for perioperative hemostasis in high-risk patients necessitating tracheostomy while on ECMO.

## Perspective statement

The application of ECMO has increased in recent years, creating a subset of patients necessitating tracheostomy with increased bleeding risk. Our data support this hybrid tracheostomy technique with the addition of a Rummel tourniquet to be a safe and effective adjunct for perioperative hemostasis in high-risk patients necessitating tracheostomy while on ECMO.

## Introduction

It is well established that patients requiring prolonged mechanical ventilation often benefit from a long-term airway, thus necessitating tracheostomy [1]. While patients are beginning to receive tracheostomy at earlier timepoints, the management of tracheostomy site bleeding complications remains a challenge for critical care medicine, ENT, and thoracic surgery teams alike. There exists a critically ill and inherently complex subset of patients requiring tracheostomy that are also on extracorporeal membrane oxygenation (ECMO); this population has grown in recent years as the utilization of ECMO in both acute respiratory distress syndrome (ARDS) and other non-traditional applications has exponentially increased [[2], [3], [4]]. As many patients on ECMO require therapeutic anticoagulation, the bleeding risk associated with tracheostomy is remarkably high. These risks were clearly demonstrated during the height of the COVID-19 pandemic as the number of patients requiring both long-term mechanical ventilation and ECMO significantly increased [[5], [6], [7]].

This complex population of patients on ECMO necessitating tracheostomy posed an interesting set of challenges, particularly the management of perioperative bleeding risk [8,9]. In an effort to safely perform bedside tracheostomy in this patient population, our institution employed a hybrid dilational tracheostomy technique utilizing a Rummel tourniquet. The addition of a Rummel tourniquet allowed for enhanced perioperative hemostasis in our experience. While this technique was developed out of necessity during the COVID-19 pandemic, we believe it is a useful adjuvant to reduce bleeding risk for all patients on ECMO necessitating tracheostomy.

## Methods

### Patient selection

A total of 24 patients on ECMO underwent bedside hybrid tracheostomy from 06/2020 to 01/2022 (Table 1). Inclusion criteria for the study were patients who had been admitted to the Mayo Clinic Hospital, placed on V—V ECMO and subsequently underwent a bedside hybrid tracheostomy procedure in the intensive care unit negative pressure room. The majority of these patients were COVID-19 positive. All procedures were performed by the same operating surgeon (JD) and the procedure itself was highly protocolized for purposes of consistency. This study was reviewed by the COVID-19 research committee as well as the traditional IRB committee who determined this study was exempt.

**Table 1:** Patient demographics and clinical details.

Age, Gender	Ethnicity	BMI	Preoperative ventilator days	Preoperative ECMO days	Total ECMO days	Tracheostomy size and type
32 F	Caucasian	27	51	48	87	8 Fr Shiley
42 M	Caucasian	32	41	39	46	8 Fr Shiley
33 F	Caucasian	36	55	3	10	8 Fr Shiley
34 F	Caucasian	45	25	8	96	6 Fr Shiley XLT Proximal
60 M	Caucasian	29	21	5	35	8 Fr Shiley
40 M	Filipino	38	11	7	66	7 Fr Shiley XLT Proximal
45 M	Hispanic	25	5	4	73	6 Fr Shiley
47 M	Caucasian	40	12	8	20	6 Fr Shiley XLT Proximal
32 F	Native American	31	32	26	70	6 Fr Shiley XLT Proximal
50 F	Caucasian	36	2	1	11	6 Fr Shiley XLT Proximal
64 F	Hispanic	33	7	6	76	6 Fr Shiley XLT Proximal
32 M	Native American	35	16	16	92	6 Fr Shiley XLT Proximal
55 M	Caucasian	29	3	2	40	8 Fr Shiley
31 F	African American	31	17	8	79	6 Fr Shiley XLT Proximal
47 F	African American	57	25	21	24	8 Fr Shiley
41 M	Native American	29	22	17	42	6 Fr Shiley
39 F	Caucasian	34	10	9	26	6 Fr Shiley
22 F	Caucasian	40	12	8	28	6 Fr Shiley XLT Proximal
26 F	Native American	29	21	8	53	6 Fr Shiley XLT Proximal
51 M	Other	31	22	9	48	7 Fr Shiley
36 M	Hispanic	26	13	5	73	8 Fr Shiley
43 F	Caucasian	34	12	9	93	6 Fr Shiley XLT Proximal
47 M	Caucasian	37	10	9	35	6 Fr Shiley XLT Proximal
50 M	Caucasian	40	10	6	41	6 Fr Shiley XLT Proximal

Abbreviations: F, Female; M, Male; BMI, Body Mass Index; ECMO, Extracorporeal Membrane Oxygenation; Fr, French.

### Outcomes and measures

The primary outcome of this study was post-tracheostomy bleeding, assessed at bedside by the Critical Care Medicine and Cardiothoracic Surgery services from the time of intervention until discharge or death. Particular attention was given to perioperative anticoagulation management, the time from endotracheal intubation to tracheostomy, as well as time on ECMO prior to tracheostomy (Table 2).

**Table 2:** Perioperative details and outcomes.

Mean preoperative ventilator days	19.0
Mean preoperative ECMO days	11.8
Mean total ECMO days	52.7
Mean time AC held preoperatively, hours	4.33
Mean time AC resumed postoperatively, hours^a^	5.2
Mean EBL, ml	2.75
Postoperative bleeding	4/24 (16.67 %)

Abbreviations: ECMO, Extracorporeal Membrane Oxygenation; AC, Anticoagulation; EBL, Estimated Blood Loss.

a Single outlier of 148.5 h removed from statistical analyses as GI bleed was unrelated to tracheostomy procedure.

## Procedure and protocol

### Prior to the procedure

**Fig. 1:**
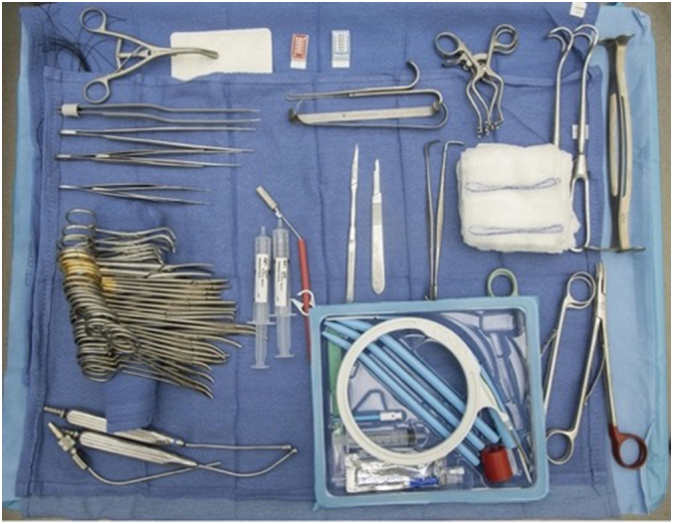
Pre-procedural tray setup.

In anticipation of the procedure, several steps were taken. A formal surgical “timeout” was performed. For patients on bivalirudin drips, it was held two to four hours prior to the start of the procedure. In their ICU room, patients were positioned as high as possible on the bed. The neck was placed into extension and a shoulder roll was utilized. The bronchoscopy tower and screen were placed to the left of the patient as close to the head as possible. Immediately prior to starting the operation, the patient was placed on 100 % fraction of inspired O2 (FiO2) through the ventilator and the ECMO circuit. At the beginning of the procedure, the FiO2 was reduced to 40 %. Of note, this was adjusted throughout the duration of the procedure as needed to maintain adequate oxygenation. The procedural tray was inspected to ensure all required instruments were available (Fig. 1).

### Procedure

The initial step of the procedure included utilizing a flexible bronchoscopy from the head of the bed. This was utilized for anatomic assessment as well as removing any excessive airway secretions. There was sterile prep and draping of the neck and chest. Prophylactic antibiotics were administered if the patient was not on antibiotic treatment. The operation began by making a 2–4 cm (cm) transverse incision approximately two finger breadths below the thyroid cartilage, or at the midpoint between the cricoid cartilage and sternal notch. Electrocautery was then used to dissect the subcutaneous tissue down to the level of the platysma which was then transected transversely. The dissection was then carried down to the linea alba cervicalis, taking care to avoid the anterior jugular veins bilaterally. If these were encountered, they were suture ligated using monofilament suture. Once the linea alba cervicalis was identified in the midline, it was divided vertically. The strap muscles were retracted laterally for adequate exposure. If the thyroid isthmus was encountered, it was retracted with a peanut retractor or divided with electrocautery to better expose the underlying trachea. At this point in the operation, the operating surgeon communicated with the bronchoscopist to reintroduce the bronchoscope back into the airway. The bronchoscope was then advanced to the level of the endotracheal (ET) tube cuff which was then taken down under direct visualization and the ET tube was withdrawn until it was positioned one to two centimeters proximal to the desired tracheostomy site. 1 ml of air was used to partially reinflate the ET tube cuff, as this affords about 100 ml of tidal volume for ventilatory purposes during this time.

**Fig. 2:**
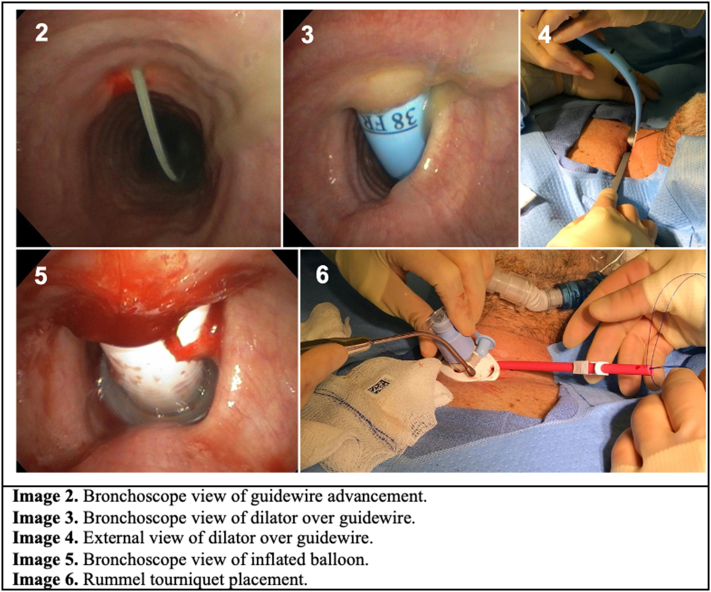
Procedural images of bedside dilational tracheostomy with Rummel tourniquet.

A 21G Finder needle was then used to identify the most appropriate location for the tracheostomy. This was visualized both from the operative field as well as bronchoscopically. After the appropriate location was determined with the 21G Finder needle, it was replaced by a 15 gauge needle with the bevel facing the distal airway. A guidewire was threaded into the airway and advanced into the proximal mainstem bronchus (Fig. 2, Image 1). There was meticulous attention to not allow the guidewire to pass into the distal airway and potentially injure the parenchyma of the lung and cause bleeding. Thus, Seldinger technique and bronchoscopic visualization were absolutely critical components of the procedure. The needle was then removed, and the guidewire left in place. A 14 Fr small dilator was passed over the wire a total of three times and subsequently removed with all of the steps being aided under bronchoscopic guidance. The Blue rhino dilator was then passed over the wire (Fig. 2, Image 3) and advanced until the skin level mark on the dilator could be visualized in the airway with the bronchoscope (Fig. 2, Image 4). The tracheostomy tube was inserted into the trachea until the cuff could be fully visualized within the lumen (Fig. 2, Image 5). The bronchoscope was pulled out of the ET tube and placed through the tracheostomy tube appliance to ensure it was correctly positioned within the trachea. The inner cannula was inserted into the tracheostomy, the balloon inflated, and the patient connected to the ventilator circuit. The bronchoscope was returned to the ET tube to ensure the tracheostomy appliance sat well within the airway and the balloon was appropriately positioned. A total of 3 ml of epinephrine mixture in a 10 ml syringe with 7 ml of air behind it (1 amp of epinephrine in 20 ml of normal saline) was flushed through the bronchoscope into the subglottic space to aid in local hemostasis.

A pursestring suture was placed around the tracheostomy stoma using 0 Prolene suture starting at the 1 o'clock position. A Rummel tourniquet was then placed and snared for postoperative hemostasis (Fig. 2, Image 6). Two 2–0 Prolene sutures were loosely placed to secure the tracheostomy in place in the 10 o'clock and 2 o'clock positions and tracheostomy ties were placed.

The bronchoscope was then reinserted back into the ET tube and the airway inspected for hemostasis. If bleeding was observed, and additional application of epinephrine saline solution was instilled. Once hemostasis was achieved, the ET tube was removed under direct bronchoscopic visualization. Finally, toilet bronchoscopy was performed through the tracheostomy.

### Post-procedure management

Following the procedure, a chest x-ray was obtained. The Rummel tourniquet was left engaged. This was evaluated daily and was left tightened typically for 24 to 48 h postoperatively. On POD#2 the Rummel was relaxed for 4–6 h at a time. The bivalirudin drip was restarted on a case dependent basis, usually within four to six hours post procedure. The bivalirudin drip was started at a low fixed dose and titrated back to the previous therapeutic range. Finally, the stay sutures were removed seven days after the procedure. The 0-prolene suture was left in place for several weeks in case needed.

## Results

### Population

From 06/2020 to 01/2022, a total of 24 patients underwent a bedside hybrid tracheostomy procedure. There was a total of twelve females and twelve males with an average age of 41.6 years (range 22–64 years). The average BMI on admission was noted to be 34 (median 34, range 25–57).

### Clinical outcomes

The primary outcome of this study, defined as bleeding from the tracheostomy site following bedside hybrid tracheostomy, occurred in four of the 24 participants (16.67 %). In each of these four cases, the bleeding was appreciated after the Rummel tourniquet had been loosened and was managed by tightening the Rummel tourniquet and packing with hemostatic agents. The Rummel was re-engaged for at least 24 h prior to re-evaluation and subsequent loosening of the tourniquet. There were no complications requiring operative intervention. Additionally, there were no stoma-related complications from the Rummel tourniquet or other aspects of the procedure. The average time from ET intubation to tracheostomy was 19 days. The average time patients were on ECMO prior to the tracheostomy was 11.8 days, while the average total time on ECMO was 52.7 days. Of particular relevance to the primary outcome was the management of perioperative anticoagulation. All patients included in the study were anticoagulated on bivalirudin prior to tracheostomy as is the practice at our institution. The mean time held pre-operatively was 4.33 h. The mean time anticoagulation was held post-operatively was 5.2 h. There was a single outlier whose anticoagulation was held for 148.5 h following the procedure secondary to an ongoing gastrointestinal bleed – this patient was excluded from statistical analysis.

### Adverse events

There were eight deaths (33.3 %) within the study group, seven of which were prior to ECMO decannulation. All deaths were attributed to complications secondary to COVID-19. The rate of postoperative peristomal hemorrhage was noted to be 16.67 % (4/24 patients). Three of the four patients experienced hemorrhage on postoperative day (POD) 2 after the Rummel tourniquet had been loosened. A single patient experienced intermittent peristomal bleeding between POD 10 and 20, again after the Rummel was loosened on POD#2. Hemostasis was achieved by packing the wound with hemostatic agents as well as tightening of the previously placed Rummel tourniquet. No operative intervention was required in any of the patients. Additionally, we did not observe any local peristomal ischemia or necrosis in patients that required re-engagement of the Rummel tourniquet.

## Discussion

It is well understood that patients requiring mechanical ventilation for an extended period of time benefit from a long-term airway [1,2,4]. While tracheostomy is a necessity in such patients, management of bleeding tracheostomy sites remains a challenge for a wide array of specialties ranging from critical care to thoracic surgery. Further complicating the issue are those requiring tracheostomy while on ECMO as therapeutic anticoagulation has been traditionally required in this population (most often unfractionated heparin or direct thrombin inhibitors such as bivalirudin) [10]. The exponential increase of ECMO utilization in recent years has driven a drastic rise in this subset of patients with an exceptionally higher bleeding risk. In the era of the COVID-19 pandemic, our institution experienced a marked increase in the number of patients necessitating tracheostomy; as the pandemic progressed, this population grew to include a significant number of patients on ECMO. In an effort to minimize post-operative bleeding risk in this highly complex and critically ill patient population, we developed a hybrid dilational tracheostomy technique utilizing a Rummel tourniquet. Our data support this approach as being both safe and effective. While the majority of patients included in this study were COVID-19 positive, we believe that this technique has the potential for application in the vast majority of patients on ECMO necessitating tracheostomy, irrespective of COVID status.

While recent advancements in ECMO circuit technology has enabled some centers to reduce anticoagulation in ECMO patients to that of standard DVT prophylaxis, bivalirudin use remains the standard of practice at our institution, particularly in patients with severe COVID-19 due to the associated hypercoagulability [11]. As such, particular attention was paid to the mean time anticoagulation was held preoperatively (mean 4.33 h) and postoperatively (mean 5.2 h). Of note, a single patient was excluded from postoperative anticoagulation statistics in the setting of ongoing gastrointestinal bleeding. Postoperative anticoagulation was held for 148.5 h in this instance; when included in calculations, the mean time until resuming anticoagulation postoperatively is 11.5 h (median 6 h). In each of our patients, anticoagulation was restarted without bolus at a low dose fixed rate for 12 h prior to advancement to full therapeutic dosing. Based on these limited data, we found that holding anticoagulation for 4–5 h both pre- and post-operatively appears to be safe without significantly increasing the risk of perioperative hemorrhage.

Our hybrid technique was performed at bedside in negative pressure rooms at a single institution. In an attempt to more adequately manage perioperative bleeding in this population, we utilized a pursestring suture around the tracheostomy stoma with the addition of a Rummel tourniquet [10]. Post-operative hemorrhage was observed in four of the 24 patients included in the study (16.67 %). Three of these patients experienced peristomal bleeding on POD 2 and the bleeding was appreciated after the Rummel tourniquet had been loosened. In each of these cases, the peristomal bleeding was managed by tightening of the Rummel tourniquet and application of hemostatic agents at the bedside. The fourth patient experienced intermittent peristomal bleeding between POD 10 and POD 20. In this instance, the Rummel tourniquet was re-engaged on multiple occasions with subsequent resolution of bleeding. All patients were managed at the bedside with no operative intervention. As was demonstrated in this population, the utilization of hemostatic packing agents and tightening of the tourniquet enabled hemostasis without any additional procedural interventions. The use of a pursestring suture at the skin is associated with increased risk of skin bleeding, however this was mitigated with the simple application of surgical skin glue at procedure close. In our population, we did not note any associated wound complications or skin necrosis associated with the pursestring suture.

We recognize that the greatest limitation of this study lies in the lack of a matched control group. While a direct comparison of outcomes in patients undergoing tracheostomy while on ECMO would be beneficial, we did not feel it was ethical to stratify critically ill patients to a treatment arm without Rummel tourniquet when there appeared to be obvious benefit to the patient with minimal associated risk. While we cannot offer a direct comparison group, rates of bleeding with tracheostomy in patients on ECMO has been cited between 25 % and 40 % in the literature [4,[12], [13], [14]]. It is feasible to consider that without the utilization of a Rummel tourniquet, many of these cases may have necessitated operative intervention. Additionally, we note that our patient population is comprised largely of COVID-19 patients. Recent data has demonstrated that bleeding rates in COVID-19 patients undergoing tracheostomy while on ECMO are even higher than their non-ECMO counterparts, thus providing further support for the potential benefit of a Rummel tourniquet.^13,15^ The utilization of a Rummel tourniquet for hemostatic control in all ECMO patients necessitating tracheostomy has become standard at our institution with promising initial results.

In summary, we have found that our bedside hybrid dilational tracheostomy technique with the addition of a Rummel tourniquet can be employed safely and aid in the management of perioperative hemorrhage for several reasons. The initial open dissection of the subcutaneous tissues down to the level of the trachea allows for adequate hemostasis. This contrasts with the percutaneous tracheostomy method which may pose an increased bleeding risk in the setting of anticoagulation. The dilational component of our hybrid tracheostomy technique, which is the preference of the operating surgeon to minimize the total amount of time the airway is open, also proved to be safe and effective. There were no cases of trauma to the airway or malpositioning of the tracheostomy. All patients were followed longitudinally and there have been no significant stomal or subglottic complications to date. Additional procedural components that we believe aid in adequate hemostasis is the addition of dilute epinephrine solution in the subglottic space as well as the Rummel tourniquet. The most novel aspect of this technique, the Rummel tourniquet, can be implemented safely and provide an incredibly valuable adjunct for hemostasis management in high-risk patients on ECMO.

## Conclusion

Our data support that our bedside hybrid dilational tracheostomy technique can be done safely in critically ill patients on ECMO without significant risk of perioperative complications. Specifically, the use of a Rummel tourniquet appears to be a very effective adjunct to hemostasis in this high-risk patient population. We believe that these data will be increasingly beneficial as the utilization of ECMO and long-term mechanical ventilation continues to grow.

## CRediT authorship contribution statement

***Britton B Donato:*** Conceptualization, methodology, formal analysis, investigation, data curation, original writing, review and editing, project administration. ***Marisa Sewell:*** formal analysis, investigation, original writing, review and editing. ***Megan Campany:*** formal analysis, data curation, review and editing, visualization. ***Ga-ram Han***: Investigation, review and editing ***Taylor S Orton***: investigation, review and editing, visualization. ***Marko Laitinen***: Investigation, review and editing, ***Jacob Hammond:*** Investigation, review and editing. ***Xindi Chen***: data curation, review and editing. ***Jasmina Ingersoll:*** Methodology, investigation. ***Ayan Sen***: Conceptualization, methodology, investigation, review and editing, supervision. ***Jonathan D'Cunha***: Conceptualization, methodology, validation, formal analysis, investigation, review and editing, supervision, project administration.

## Consent statement

Written consent was not obtained from the patients included in this study as the COVID-19 research and traditional IRB committees deemed the study exempt and all collected information was de-identified.

## Declaration of competing interest

The authors have no disclosures or conflicts of interest to disclose.
